# Roles of eIF2α kinases in the pathogenesis of Alzheimer’s disease

**DOI:** 10.3389/fnmol.2014.00022

**Published:** 2014-04-16

**Authors:** Masuo Ohno

**Affiliations:** ^1^Nathan Kline Institute, Center for Dementia ResearchOrangeburg, NY, USA; ^2^Department of Psychiatry, New York University Langone Medical CenterNew York, NY, USA

**Keywords:** Alzheimer’s disease, elF2α, PERK, PKR, BACE1, amyloid-β, ATF4, learning and memory

## Abstract

Cell signaling in response to an array of diverse stress stimuli converges on the phosphorylation of eukaryotic initiation factor-2α (eIF2α). Evidence is accumulating that persistent eIF2α phosphorylation at Ser51 through prolonged overactivation of regulatory kinases occurs in neurodegenerative diseases such as Alzheimer’s disease (AD), leading to shutdown of general translation and translational activation of a subset of mRNAs. Recent advances in the development of gene-based strategies and bioavailable inhibitors, which specifically target one of the eIF2α kinases, have enabled us to investigate pathogenic roles of dysregulated eIF2α phosphorylation pathways. This review provides an overview of animal model studies in this field, focusing particularly on molecular mechanisms by which the dysregulation of eIF2α kinases may account for synaptic and memory deficits associated with AD. A growing body of evidence suggests that correcting aberrant eIF2α kinase activities may serve as disease-modifying therapeutic interventions to treat AD and related cognitive disorders.

## INTRODUCTION

Although the molecular cause of Alzheimer’s disease (AD) has not been completely understood, recent investigations increasingly implicate the aberrant translational machinery through the α subunit of eukaryotic initiation factor-2 (eIF2α) in the pathogenesis of this devastating neurodegenerative disease. It has been reported that eIF2α phosphorylation is significantly increased in the brains of sporadic AD patients as well as different lines of amyloid precursor protein (APP)/presenilin 1 (PS1) transgenic mice (Chang et al., 2002; Page et al., 2006; Kim et al., 2007; O’Connor et al., 2008; Devi and Ohno, 2010, 2013b; Mouton-Liger et al., 2012). Accumulation of misfolded proteins such as amyloid-β (Aβ) is known to induce eIF2α phosphorylation, whereas genetic and environmental risks for AD may be associated with modulation of the eIF2α phosphorylation pathway. The phosphorylation of eIF2α at Ser51 inhibits general translation initiation, representing a protective cellular mechanism that induces the transient shutdown of protein synthesis (UPR: unfolded protein response; Hoozemans et al., 2005; Lee et al., 2010). However, emerging evidence from animal model studies suggests that sustained eIF2α phosphorylation and translational repression of global protein synthesis, which occur under severe or prolonged stress conditions (Erguler et al., 2013), may lead to synaptic failure accompanied by reductions in vital synaptic proteins, neurodegeneration, and memory deficits associated with AD (Devi and Ohno, 2013a, under review; Ma et al., 2013).

While eIF2α phosphorylation suppresses general protein synthesis, it is shown to paradoxically cause translational activation of a subset of mRNAs, including the β-secretase called β-site APP-cleaving enzyme 1 (BACE1; De Pietri Tonelli et al., 2004; Lammich et al., 2004; Mihailovich et al., 2007; O’Connor et al., 2008; Devi and Ohno, 2010) and the transcriptional modulator activating transcription factor 4 (ATF4; Harding et al., 2000; Vattem and Wek, 2004). In accordance with persistently great amounts of phosphorylated eIF2α, expression levels of BACE1, a key enzyme responsible for triggering the production of Aβ peptides, are significantly elevated in AD brains (Fukumoto et al., 2002; Holsinger et al., 2002; Yang et al., 2003; Li et al., 2004; Ohno et al., 2007; Zhao et al., 2007; Cai et al., 2012). A recent report also demonstrates AD-related upregulation of ATF4 (Lewerenz and Maher, 2009), which is a repressor of cAMP response element binding protein (CREB)-dependent transcription critical for memory consolidation (CREB-2; Abel et al., 1998; Silva et al., 1998; Chen et al., 2003). Therefore, aberrant eIF2α phosphorylation may underlie AD pathogenesis and memory impairments not only as a downstream mechanism of Aβ accumulation but also by accelerating β-amyloidogenesis through BACE1 elevations and directly suppressing CREB function. In this article, I review recent publications suggesting multifaceted deleterious mechanisms by which dysregulated eIF2α kinases may cause memory deficits associated with AD. The findings would have important implications for the development of novel therapeutic interventions targeted at eIF2α kinases.

## eIF2α KINASES AND TRANSLATIONAL CONTROL BY eIF2α PHOSPHORYLATION

**FIGURE 1:**
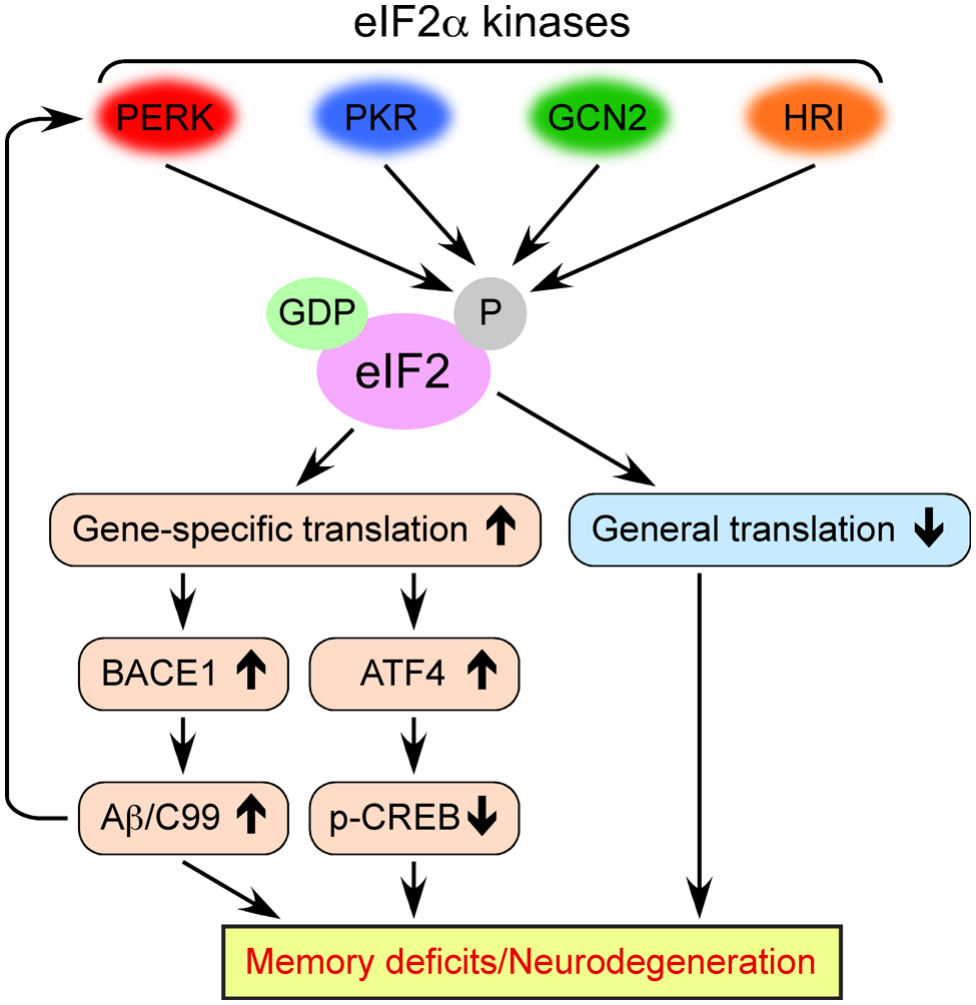
**Multiple molecular mechanisms by which dysregulated eIF2α kinase activities may lead to memory deficits and neurodegeneration associated with AD**. Four eIF2α kinases become activated in response to diverse cellular stress stimuli. Persistent eIF2α phosphorylation through aberrant activation of these kinases in AD causes the inhibition of general translation, while it activates gene-specific translation of mRNAs such as BACE1 and ATF4.

eIF2 consists of three subunits (α, β, and γ) and binds GTP and Met-tRNA_i_^Met^ (initiator methionyl-tRNA) to form a ternary complex, which delivers the initiator tRNA to the 40S ribosomal subunit. Exchange of GDP for GTP on the γ subunit is catalyzed by eIF2B, a guanine nucleotide exchange factor that is required to replenish the active GTP-bound form of eIF2 complex for a new round of translational initiation. Phosphorylation of eIF2 on its α subunit at Ser51 in response to diverse stress stimuli suppresses general translation initiation, since it converts eIF2 to a competitive inhibitor of eIF2B by blocking the GDP-GTP exchange reaction and reducing the dissociation rate of eIF2 from eIF2B (**Figure 1**). Although phosphorylation of eIF2α causes a reduction in general translation, it also selectively increases the translation of a subset of mRNAs that contain upstream open reading frames (uORFs). The molecules that undergo the gene-specific translational upregulation via eIF2α phosphorylation include the β-secretase enzyme BACE1 (De Pietri Tonelli et al., 2004; Lammich et al., 2004; Mihailovich et al., 2007; O’Connor et al., 2008; Devi and Ohno, 2010) and the CREB repressor ATF4 (CREB-2; Harding et al., 2000; Vattem and Wek, 2004), which are closely associated with the development of AD pathology and deficient memory formation (**Figure 1**). The molecular mechanism of eukaryotic translation initiation and its regulation have been described in detail in previous reviews (Costa-Mattioli and Sonenberg, 2008; Costa-Mattioli et al., 2009; Jackson et al., 2010; Donnelly et al., 2013).

The phosphorylation of eIF2α at Ser51 is controlled by four protein kinases such as general control non-derepressible-2 kinase (GCN2), double-stranded RNA-activated protein kinase (PKR), PKR-like endoplasmic reticulum kinase (PERK), and heme-regulated inhibitor kinase (HRI; **Figure 1**; Costa-Mattioli and Sonenberg, 2008; Donnelly et al., 2013). The eIF2α kinases except for HRI are prominently expressed in the mammalian brain. These eIF2α kinases share a conserved kinase domain but have divergent regulatory domains to specifically become activated (i.e., phosphorylated) in response to a variety of cellular stress stimuli, as reviewed previously (Donnelly et al., 2013). In brief, PERK is primarily activated by the accumulation of misfolded proteins in the endoplasmic reticulum (ER), a phenomenon termed ER stress. Generally, PERK-dependent eIF2α phosphorylation is thought to block global translation initiation and in this manner can alleviate ER stress by reducing the amount of protein transport into the ER. This allows the ER time to refold misfolded proteins and dispose of those that are terminally misfolded, an important element of cellular protective UPR processes. PKR was initially discovered as a kinase that phosphorylates eIF2α in response to viral infection or double-stranded RNA, thereby blocking the translation of viral mRNAs and promoting apoptosis. GCN2 is primarily a sensor of amino acid availability and a regulator of changes in gene expression in response to amino acid deprivation. GCN2 is also activated by UV irradiation and viral infection. HRI is predominantly found in erythroid cells and activated by heme deficiency. Remarkably, considerable evidence is accumulating that these eIF2α kinases are dysregulated on different disease conditions and may play pathogenic roles, especially, in neurodegenerative disorders including AD and related cognitive impairments, as summarized in this review.

## eIF2α PHOSPHORYLATION AND AD

It has become increasingly apparent that amounts of phosphorylated eIF2α are significantly elevated in sporadic AD brains (Chang et al., 2002; Hoozemans et al., 2005; Kim et al., 2007; O’Connor et al., 2008; Mouton-Liger et al., 2012; Natunen et al., 2013; Segev et al., 2013). Moreover, the elevation in eIF2α phosphorylation is successfully recapitulated in different transgenic mouse models of AD that exhibit memory impairments, including 5XFAD (O’Connor et al., 2008; Devi and Ohno, 2010, 2013a,b ), Tg2576 (Kim et al., 2007), and APP/PS1 KI (Page et al., 2006; Mouton-Liger et al., 2012). Among the eIF2α kinases, these studies detect aberrant activation of PERK and/or PKR in brain, which may thus represent major mediators of eIF2α phosphorylation that overly occurs with relevance to AD.

Interestingly, recent animal model studies also reveal that genetic and environmental risk factors for sporadic AD are associated with increased eIF2α phosphorylation. For example, amounts of phosphorylated eIF2α increase with normal aging in wild-type mice, while mice overexpressing human apolipoprotein E4 (ApoE4: a strong genetic risk factor that modulates the prevalence, age of onset and the burden of pathology in sporadic AD) show elevations in eIF2α phosphorylation in brains and deficient learning and memory, as compared with age-matched ApoE3 control mice (Segev et al., 2013). This ApoE4-related eIF2α phosphorylation occurs at an early age (4 months) concomitant with overactivation of PKR and GCN2 pathways and is not further enhanced with aging, suggesting that genetic and aging risks for AD converge on the eIF2α phosphorylation pathway. We have demonstrated that young (3- to 4-month-old) 5XFAD mice, which have not yet showed an increase in eIF2α phosphorylation at baseline levels, exhibit robust activation of the PERK-eIF2α pathway and enhanced Aβ plaque pathology in response to potential environmental risks for AD such as insulin-deficient diabetic conditions and behavioral stress (Devi et al., 2010, 2012). Conversely, a recent study shows that physical activity (i.e., treadmill exercise), a therapeutic regimen hypothesized to delay AD progression, prevents ER stress-related activation of PERK-dependent eIF2α phosphorylation, apoptosis, Aβ accumulation and cognitive impairments in PS2 mutant mice (Kang et al., 2013). Taken collectively, these data provide compelling evidence that the increases in eIF2α phosphorylation through dysregulated eIF2α kinase activities may be closely associated with the pathogenesis or acceleration of AD and responsible for disrupting memory processes in this disease.

## eIF2α KINASES AS NEGATIVE MODULATORS OF MEMORY FUNCTION

Recent work suggests the importance of translational control through eIF2α phosphorylation in learning and memory. First, Costa-Mattioli et al. (2005) have reported that GCN2 deficiency facilitates long-term memory formation as well as hippocampal late-phase long-term potentiation (L-LTP: a long-lasting synaptic plasticity model for memory consolidation) when they are induced by a weak subthreshold training or tetanic stimulation protocol that does not normally elicit stable long-term memory or L-LTP. Similarly, the suppression of eIF2α phosphorylation in heterozygous knock-in mice with an eIF2α^+^^/S51A^ point mutation also enhances L-LTP and memory formation in multiple learning paradigms such as the Morris water maze, fear conditioning and conditioned taste aversion (Costa-Mattioli et al., 2007). These results are consistent with the observations that behavioral training for contextual fear conditioning and novel taste learning tasks results in reduced levels of eIF2α phosphorylation at Ser51 in the hippocampus, whereas hippocampal application of Sal003, an inhibitor of eIF2α dephosphorylation, impairs not only L-LTP but also contextual and taste memories (Costa-Mattioli et al., 2007; Jiang et al., 2010; Stern et al., 2013). Moreover, baseline levels of ATF4, a repressor of CREB (CREB-2), is decreased concomitant with reduced eIF2α phosphorylation in GCN2-deficient mice, suggesting that these behavioral alterations may be associated with changes in CREB-mediated gene expression. Therefore, the findings provide convergent evidence that GCN2-dependent eIF2α phosphorylation works as a negative regulator of L-LTP and long-term memory formation through reducing not only general translation initiation but also CREB-dependent transcription. However, it should be noted that GCN2-deficient mice exhibit, conversely, declines in memory formation and L-LTP when they are exposed to a robust training or stronger tetanic stimulation protocol that is sufficient to produce stable long-term memory or L-LTP (Costa-Mattioli et al., 2005). Conceivably, stronger stimulations may potentiate an inhibitory pathway that is upregulated by the lack of translational repression in these mice, so the GCN2-mediated ATF4 translation needs to be tightly controlled for normal memory function and synaptic plasticity.

Likewise, the impacts of genetic and pharmacologic manipulations of PKR on learning and memory as well as hippocampal L-LTP have also been recently investigated. Jiang et al. (2010) developed a novel conditional transgenic mouse model in which PKR is specifically increased in hippocampal CA1 pyramidal cells by the chemical inducer. These mice show deficient L-LTP and contextual fear memory concomitant with increased levels of eIF2α phosphorylation. Notably, they found that the facilitation of PKR-dependent eIF2α phosphorylation in this model elevated ATF4 translation and suppressed CREB-dependent gene expression (e.g., BDNF: brain-derived neurotrophic factor), while it was not sufficient to affect *de novo* general translation. Therefore, it seems likely that increased gene-specific translation of ATF4 rather than global translational inhibition may be a key event to deteriorate long-term memory and L-LTP. This idea is supported by the previous observation that ATF4 deficiency was able to prevent hippocampal L-LTP suppression associated with pharmacologic induction of eIF2α phosphorylation with Sal003 (Costa-Mattioli et al., 2007). Furthermore, a recent study demonstrates that reducing eIF2α phosphorylation by pharmacologically inhibiting PKR activities in mice and rats enhances their novel taste and conditioned taste aversion memories (Stern et al., 2013).

Meanwhile, Trinh et al. (2012) recently generated a mouse model that has forebrain-specific and postnatal deletion of PERK. These PERK mutant mice show reductions in eIF2α phosphorylation and ATF4 expression but no change in general translation. Therefore, ATF expression seems more responsive than general translation to the alteration of eIF2α phosphorylation caused by PKR (Jiang et al., 2010) and PERK gene manipulations (Trinh et al., 2012). Intriguingly, although conditional PERK removal does not affect initial learning or memory formation, it results in impaired behavioral flexibility including deficient fear memory extinction and reversal learning in the Morris water maze or Y-water maze. It appears that the reversal learning as well as initial learning is normally associated with a reduction in phosphorylated eIF2α in wild-type controls. However, since mice with conditional PERK ablation show dramatically reduced levels of eIF2α phosphorylation after acquisition, they lack further reduction in response to reversal learning. Altogether, the neurobiological studies using genetic and pharmacologic manipulations of eIF2α kinases have indicated that translational regulation of ATF4 expression through eIF2α phosphorylation should be tightly controlled for normal mnemonic processing, thus suggesting the possibility that dysregulated activities of eIF2α kinases on disease conditions may account for cognitive disorders.

## eIF2α KINASES AND MEMORY DEFICITS IN AD

To directly address the molecular mechanisms by which overactivation of the eIF2α phosphorylation pathway may cause AD-associated memory impairments, recent studies have tested whether genetic manipulations of eIF2α kinases in transgenic mouse models can rescue their synaptic and cognitive failures. By crossing forebrain-specific PERK knockout mice with APP/PS1 transgenic mice, Ma et al. (2013) showed that PERK ablation prevents hippocampal eIF2α phosphorylation and memory impairments, as assessed by the hippocampus-dependent spatial learning paradigms such as the Morris water maze, Y-water maze and object location tasks. Furthermore, this report revealed that conditional PERK removal ameliorates deficient LTP (a cellular basis for learning and memory) at hippocampal Schaffer collateral-CA1 synapses in APP/PS1 mice. As observed with behavioral training, LTP-inducing high-frequency stimulation causes dephosphorylation of eIF2α, which is prevented by application of exogenous Aβ. Interestingly, Aβ-induced impairment of CA1 LTP in hippocampal slices is also rescued by deleting PERK, suggesting that PERK-dependent hyperphosphorylation of eIF2α as a consequence of Aβ accumulation may underlie deficient synaptic plasticity (Ma et al., 2013).

We recently found that PERK haploinsufficiency is sufficient to block overactivation of the PERK-dependent eIF2α phosphorylation pathway in 5XFAD transgenic mice (Devi and Ohno, under review), which represent an early onset and aggressive amyloid mouse model based on a combination of five familial AD (FAD) mutations (Oakley et al., 2006; Ohno et al., 2006, 2007). Our results demonstrate that PERK haploinsufficiency can also lead to amelioration of memory deficits in 5XFAD mice, as tested by the hippocampus-dependent contextual fear conditioning. Therefore, two independent investigations using different AD mouse models combined with PERK gene targeting approaches consistently support the idea that dysregulated PERK activities and eIF2α hyperphosphorylation account for memory deficits associated with AD.

It has also been examined whether genetic deletion of GCN2, another eIF2α kinase, may have beneficial effects on memory defects in transgenic mouse models of AD (Devi and Ohno, 2013a; Ma et al., 2013). A recent study shows that knocking out GCN2 in APP/PS1 mice rescues spatial memory deficits in the water maze task, as observed with conditional PERK deletion (Ma et al., 2013). Moreover, GCN2 gene ablation is demonstrated to prevent LTP deficits found in Aβ-applied hippocampal slices as well as in APP/PS1 mice. Therefore, GCN2 also seems to be an eIF2α kinase of which dysregulation may be responsible for synaptic and mnemonic deficits in AD. In contrast, we found that GCN2^-^^/^^-^ and GCN2^+^^/^^-^ deficiencies aggravate rather than suppress eIF2α phosphorylation in 5XFAD mice, thus failing to rescue memory deficits in the contextual fear conditioning task (Devi and Ohno, 2013a). Interestingly, our data indicate that GCN2 deletion causes further activation of the PERK-dependent eIF2α phosphorylation pathway in 5XFAD mice in the absence of changes in the PKR pathway. It should be noted that the overactivation of PERK in response to GCN2 deletion is observed specifically in 5XFAD mice, since GCN2^-^^/^^-^ mice show reduced eIF2α phosphorylation (relative to wild-type controls) without compensatory changes in phosphorylated PERK levels. Therefore, we postulate that signaling mechanisms controlling eIF2α phosphorylation are different between normal and severe AD conditions. GCN2 may be an important eIF2α kinase under the physiological condition, whereas PERK-mediated eIF2α phosphorylation becomes prominent under exposure to great amounts of misfolded proteins (e.g., robust β-amyloidosis in 5XFAD) and GCN2 may function as a negative regulator of this pathway (Devi and Ohno, 2013a). Collectively, two recent studies provide contradictory results concerning the role of GCN2 in translational dysregulation through eIF2α phosphorylation and memory impairments associated with AD. These might be accounted for by the different lines of animal models used and/or neuropathological stages that represent a key determinant for the degree of stressful conditions. In any case, further study is clearly required, given that it has not been conclusively determined whether aberrant GCN2 activation may occur in AD brains.

Meanwhile, overactivation of the PKR pathway has been well established in many models of AD and patients with AD. A preclinical longitudinal study was undertaken to evaluate the effects of a PKR inhibitor (Compound C16) in APP/PS1 mice (Couturier et al., 2012). The results indicate that PKR inhibition transiently prevents inflammation without affecting Aβ concentrations in brains. However, treatments with C16 become ineffective in reducing inflammation markers and induce a great increase in Aβ levels in APP/PS1 mice during advanced stages of disease. No beneficial effects of the PKR inhibitor on spatial memory impairments, as tested by the water maze and Y-maze paradigms, are observed in these AD model mice throughout the progression of disease.

## eIF2α KINASES AND GENERAL TRANSLATIONAL INHIBITION IN AD

The analysis of *in silico* model reveals that chronic and severe ER stress induces persistent PERK-dependent phosphorylation of eIF2α, which is sufficient to induce shutdown of translation (Erguler et al., 2013). In accordance with this scenario, it is shown that *de novo* protein synthesis is significantly suppressed concomitant with elevated levels of phosphorylated eIF2α in APP/PS1 transgenic mice, whereas conditional PERK deletion prevents the reduction of general protein synthesis in these mice (Ma et al., 2013). Moreover, knocking out PERK restores reduced levels of vital plasticity-related synaptic proteins and improves synaptic and cognitive dysfunctions in APP/PS1 mice. We recently extend these findings by showing that reducing PERK-dependent eIF2α phosphorylation in 5XFAD mice is able to prevent their AD-like cholinergic neuron loss in the medial septum (Devi and Ohno, under review). It is important to note a study demonstrating that treatments with the specific PERK inhibitor GSK2606414 prevent aberrant eIF2α phosphorylation and translational failure in prion-infected mice, leading to rescue from deficient synaptic proteins, neurodegeneration, and clinical signs of prion disease including memory impairments (Moreno et al., 2013). Taken together, these findings support the concept that the dysregulated PERK-eIF2α pathway and translational repression may be common molecular mechanisms underlying neurodegenerative diseases that occur as a consequence of the accumulation of misfolded proteins. However, two other pathways of the UPR (i.e., ATF6 and IRE1α) are not activated in APP/PS1 or prion-diseased mice (Ma et al., 2013; Moreno et al., 2013), and it currently remains unclear whether PERK overactivation found in AD animal models may be part of the UPR signaling (Endres and Reinhardt, 2013).

## eIF2α KINASES AND BACE1 ELEVATION IN AD

As opposed to the shutdown of general translation, dysregulated eIF2α phosphorylation leads to translational upregulation of the β-secretase enzyme BACE1. O’Connor et al. (2008) reported that levels of phosphorylated eIF2α are significantly elevated in human AD brains and positively correlate with BACE1 expression levels and Aβ plaque loads. Similarly, phospho-eIF2α-related increases in BACE1 protein are also found in brains of 5XFAD model mice (O’Connor et al., 2008; Devi and Ohno, 2010) in the absence of changes in its mRNA levels (Zhao et al., 2007). Therefore, it seems likely that the BACE1 elevation is not the results of increased BACE1 gene transcription or mRNA stability at least in this AD model. Remarkably, we previously demonstrated that increased amounts of phosphorylated eIF2α resulting from the application of Sal003, a specific inhibitor of its phosphatase, can elevate BACE1 protein levels in young 5XFAD mice, which have not yet showed BACE1 upregulation consistent with only marginal changes in eIF2α phosphorylation (Devi and Ohno, 2010). Sal003 also increases BACE1 expression and Aβ production in primary neurons (O’Connor et al., 2008). Together, these findings provide convergent evidence for a link between aberrant eIF2α phosphorylation and BACE1 elevation in AD.

Which eIF2α kinase(s) may be responsible for mediating BACE1 upregulation? We recently demonstrate that reducing PERK-dependent eIF2α phosphorylation blocks BACE1 elevation in advanced stages of 5XFAD mice (8-to 9-month-old), leading to the suppression of β-amyloidogenesis as evidenced by decreased levels of the β-cleaved C-terminal fragment of APP (β-CTF or C99), Aβ40 and Aβ42 peptides, and amyloid plaque burden in PERK^+^^/^^-^·5XFAD mice (Devi and Ohno, under review). APP/PS1 mice with conditional PERK ablation also have reduced levels of Aβ and C99 (Ma et al., 2013). Furthermore, a recent report shows that administration of arctigenin, a natural product from *Arctium lappa* (L.), to APP/PS1 mice can block the translational upregulation of BACE1 by suppressing the PERK-eIF2α pathway in the absence of transcriptional alteration (Zhu et al., 2013). This action of arctigenin leads to improved memory performances of APP/PS1 mice in the water maze concomitant with reduced Aβ production and plaque loads, although enhanced Aβ clearance through autophagy attributable to mTOR inhibition is also noted. Conversely, GCN2-deficient 5XFAD mice exhibit the facilitation of PERK-dependent eIF2α phosphorylation, which is accompanied by the exacerbation of BACE1 elevation, Aβ/C99 accumulation, and plaque pathology (Devi and Ohno, 2013a). Furthermore, we found that BACE1 is elevated concomitant with robust activation of the PERK-eIF2α pathway in young 5XFAD mice under exposure to insulin-deficient diabetic conditions or behavioral stress (Devi et al., 2010, 2012). In accordance with these observations in transgenic mouse models, transfection with dominant negative PERK, but not dominant negative GCN2, prevents energy deprivation-induced phosphorylation of eIF2α and BACE1 elevation in an *in vitro* model of incipient AD conditions (O’Connor et al., 2008). Therefore, literature seems consistent with the pathogenic role of dysregulated PERK in mediating robust eIF2α phosphorylation that accounts for BACE1 elevation and the consequent acceleration of neurotoxic Aβ/C99 accumulation associated with AD.

The PKR-eIF2α phosphorylation pathway is also shown to be highly activated in AD brains and correlate with the degree of BACE1 elevation (Mouton-Liger et al., 2012). In this study, APP/PS1 KI mice have similarly increased levels of BACE1 expression concomitant with overactivation of PKR-eIF2α signaling. Mechanistically, it is demonstrated that BACE1 elevation occurs in response to hydrogen peroxide-induced oxidative stress in cell culture, which is blocked by a specific inhibitor or siRNA targeting PKR (Mouton-Liger et al., 2012). Moreover, *in vivo* and cell culture experiments reveal that infection with herpes simplex virus type 1 (HSV1: a virus suggested to be implicated in AD development) causes activation of the PKR-eIF2α pathway, resulting in elevations of BACE1 expression and Aβ/C99 generation (Ill-Raga et al., 2011). A recent study also reports that pharmacological inhibition of PKR successfully blocks BACE1 upregulation in APP/PS1 transgenic mice; however, these effects do not necessarily lead to cerebral Aβ reduction or cognitive benefits (Couturier et al., 2012). Interestingly, we found that 5XFAD mice, an aggressive AD model in which PERK-dependent BACE1 elevation is prominent (Devi and Ohno, under review), do not show activation of the PKR-eIF2α phosphorylation pathway (Devi and Ohno, 2013a, b). Collectively, it seems likely that the severity of disease or different stress conditions to which nerve cells are exposed during the course of AD development may be an important factor to determine the predominant eIF2α kinase(s) that may be responsible for BACE1 elevation.

It has been demonstrated that Aβ accumulation induces BACE1 elevation in neurons (most likely, swollen dystrophic neurites) surrounding plaques, which in turn further accelerates Aβ generation in 5XFAD mouse and human AD brains (Zhao et al., 2007; Zhang et al., 2009; Devi and Ohno, 2013a; Kandalepas et al., 2013). Therefore, it is conceivable that eIF2α kinases (especially, PERK and PKR) may be mediators of amyloid plaque growth. Likewise, it is important to note that both PERK and PKR are also involved in tau hyperphosphorylation. These eIF2α kinases activate glycogen synthase kinase-3β (GSK-3β), a major tau kinase, in brain (Baltzis et al., 2007; Bose et al., 2011). Furthermore, there is evidence that PERK and PKR may also facilitate phosphorylation of tau independently of GSK-3β connection in some experimental setting (Azorsa et al., 2010; Ho et al., 2012). Of particular interest, Ho et al. (2012) report that ER stress-related activation of the PERK-eIF2α pathway causes tau phosphorylation, while levels of phosphorylated PERK and eIF2α are increased in response to experimentally induced hyperphosphorylation of tau in primary cortical cultures and Tau^P301L^ transgenic mice. Taken together, it is likely that the dysregulation of eIF2α kinases may represent a crucial signaling component underlying the development of both plaque and tangle pathologies through interplays with BACE1/Aβ and tau phosphorylation, respectively.

## eIF2α KINASES AND ATF4 ELEVATION IN AD

ATF4, a repressor of CREB (CREB-2), is another signaling molecule of which translation is facilitated by eIF2α phosphorylation (Harding et al., 2000; Vattem and Wek, 2004). As described above, there is compelling evidence that ATF4 negatively regulates memory processes by suppressing CREB activity and that level of ATF4 and phosphorylated eIF2α are upregulated and tightly correlated each other in AD brains (Lewerenz and Maher, 2009). Therefore, it is important to understand signaling mechanisms underlying these changes. Our recent work demonstrates that 5XFAD mice recapitulate increased levels of ATF4 expression and CREB dysfunction concomitant with robust elevation of phosphorylated eIF2α (Devi and Ohno, under review). Remarkably, we found that PERK haploinsufficiency can abolish ATF4 elevation and rescue deficient CREB function in 5XFAD mice. This is consistent with the results showing that reducing eIF2α phosphorylation with conditional PERK deletion in APP/PS1 mice blocks ATF4 upregulation (although CREB signaling is not studied in this report; Ma et al., 2013). Conversely, enhanced PERK-dependent eIF2α phosphorylation in GCN2-deficient 5XFAD mice leads to the aggravation of ATF4 elevation and CREB dysfunction (Devi and Ohno, 2013a). Although further investigation is needed to test the role of other eIF2α kinases, the current data strongly suggest that the PERK-eIF2α pathway is critically involved in mediating ATF4-dependent CREB dysfunction associated with AD.

## CONCLUDING REMARKS

The findings summarized in this review provide experimental evidence that the aberrant activation of eIF2α phosphorylation pathways found in AD may be responsible for multifaceted memory-deteriorating and neurodegenerative mechanisms, including accelerated β-amyloidogenesis through BACE1 elevation, CREB dysfunction via ATF4 upregulation, and inhibition of general translation (**Figure 1**). It should be noted that cross-talk impacts such as BACE1- or Aβ-dependent suppression of CREB function (Vitolo et al., 2002; Chen et al., 2012) would worsen the detrimental outcomes in this scenario. Moreover, eIF2α kinase dysregulation may also be associated with tau hyperphosphorylation, although underlying mechanisms remain to be fully understood. As upstream signaling components, the activity of each eIF2α kinase (except for HRI) seems to play roles in controlling the deleterious events, more or less, depending upon stress conditions during the progression of AD. Challenging questions in the future are how we can determine the eIF2α kinase that is central to these detrimental mechanisms at different AD stages and whether inhibiting a single eIF2α kinase may be sufficient to exert beneficial effects in clinical settings. Currently, selective and bioavailable inhibitors of PERK (Axten et al., 2012; Moreno et al., 2013) and PKR (Couturier et al., 2010, 2012) are developed for neurodegenerative disease therapy, although their potential adverse effects (e.g., hyperglycemia with PERK inhibitors) need to be carefully addressed. Clearly, much work remains for validation and practical application, but the present data warrant further preclinical evaluations of the eIF2α kinase inhibitors in animal models (e.g., the regimen for timing and duration of drug administration to optimize their efficacies during neuropathological development) as novel disease-modifying therapeutic interventions to treat AD and related cognitive impairments.

## Conflict of Interest Statement

The author declares that the research was conducted in the absence of any commercial or financial relationships that could be construed as a potential conflict of interest.
